# Transcriptomic Profiling and WGCNA Identify ALOX5 as a Key Regulator of Iron Metabolism and Immune Crosstalk in Venous Thromboembolism

**DOI:** 10.3390/cimb48060607

**Published:** 2026-06-10

**Authors:** Zhiyun Cheng, Ruyu Bai, Yong Diao

**Affiliations:** School of Medicine, Huaqiao University, Quanzhou 362021, China; chengzhiyun@hqu.edu.cn (Z.C.); bairuyu@hqu.edu.cn (R.B.)

**Keywords:** venous thromboembolism, iron metabolism, *ALOX5*, diagnostic signature, endothelial dysfunction

## Abstract

Venous thromboembolism (VTE) is a major cause of morbidity and mortality, underscoring the need for new molecular markers to enable early detection and clarify underlying mechanisms. Iron metabolism is linked to oxidative stress, endothelial injury, and inflammation, all central to thrombosis, yet its transcriptomic contribution to VTE remains unclear. We analyzed gene expression profiles from GSE19151 and GSE48000 using differential expression and weighted gene co-expression network analysis (WGCNA), integrating results with an iron metabolism gene set. Three hub genes were identified, arachidonate 5-lipoxygenase (*ALOX5*), Rho GTPase activating protein 1 (*ARHGAP1*), and glucose-6-phosphate dehydrogenase (*G6PD*), all downregulated in VTE. Gene set enrichment indicated that *ALOX5* is involved in endothelial regulation, lipid metabolism, and immune pathways. A three-gene signature showed high diagnostic accuracy (AUC = 0.924 in the discovery cohort; 0.705 in validation). Immune deconvolution revealed broad immune remodeling and associated *ALOX5* with multiple immune cell subsets, especially M0 macrophages, and with regulators such as *TGFB1* and *IL6R*. Western blot analysis further showed that ALOX5 protein expression was significantly increased in LPS-activated HUVECs, supporting its involvement in inflammatory endothelial injury. DrugBank screening identified 19 approved drugs targeting *ALOX5*, supporting its potential for mechanistic and clinical investigation.

## 1. Introduction

Venous thromboembolism (VTE), encompassing deep vein thrombosis (DVT) and pulmonary embolism (PE), remains a critical global health challenge as a primary contributor to cardiovascular morbidity and mortality, representing a substantial portion of preventable deaths worldwide [1,2,3]. Even though we have made notable progress in diagnostic imaging and new, more effective anticoagulants are being applied, VTE still brings clinical difficulties. The manifestation of this illness often has no clear specificity; therefore, we must adopt multi-step plans to confirm the diagnosis. Moreover, even after patients receive suitable treatment, the risk of recurrence is still very high [4,5,6]. Hence, there is strong interest in searching for blood-based biomarkers; these substances can enhance early detection, assist in dividing patients into different risk groups, and help us understand more about the in-depth pathophysiological mechanisms [7].

The traditional causal explanation of VTE is derived from Virchow’s triad: venous stasis, endothelial injury, and hypercoagulability [8]. In addition to these well-known factors, recent studies pay high attention to the concept of inflammation-induced thrombosis, or “immunothrombosis”; thus, innate and adaptive immune cells interact with platelets and the coagulation system, and inflammatory mediators change the microenvironment of the venous wall [9,10,11]. Mechanisms including neutrophil extracellular trap formation and monocyte/macrophage phenotypic change have linkages with thrombus initiation, growth, and resolution [12,13,14]. Therefore, these interaction phenomena between immune and coagulation systems inform us that gene expression analysis may detect disease-related immune remodeling and could lead us to explore new possible biomarkers [15,16].

Iron metabolism is a biologically plausible pathway in VTE, but it is relatively under-investigated. Iron homeostasis impacts oxidative stress equilibrium, lipid peroxidation processes, mitochondrial activity, and immune signaling, all of which are directly linked to endothelial biology and hemostatic mechanisms [17,18,19,20]. Dysregulated iron metabolism can lead to iron-dependent oxidative damage, endothelial activation, leukocyte migration, and platelet hyperreactivity, thereby intersecting with molecular pathways associated with thrombogenesis [21,22,23]. While initial findings suggest that disruptions in iron regulation may be linked to thrombotic disorders [24], the full scope of iron metabolism-related changes in gene expression in VTE and their possible clinical relevance are still mostly unexplored.

To systematically identify potential biomarkers for VTE, the present study employed a multi-step analytical approach. Initially, differential gene expression analysis was integrated with WGCNA on a discovery cohort to generate VTE-associated molecular signatures. These candidate signatures were then intersected with a manually curated database of iron metabolism-related genes to identify potential hub genes. Finally, the diagnostic utility of these hub genes was validated on an independent validation dataset to assess their clinical applicability. The analysis of biological functions was carried out via gene set enrichment analysis (GSEA). The infiltration of immune cells was evaluated by means of Cell-type Identification By Estimating Relative Subsets Of RNA Transcripts (CIBERSORT), and the immune correlations were assessed using the Tumor and Immune System Interaction Database (TISIDB). Potential therapeutic targeting was analyzed via DrugBank, and the expression of the key hub gene was validated experimentally with an LPS-induced endothelial injury model. This research brings forward an externally verified gene marker linked with iron metabolism and VTE; therefore, we place it in the immune–endothelial interaction framework to guide future mechanism-based and translation-related research investigations.

## 2. Materials and Methods

### 2.1. Data Acquisition

**Figure 1:**
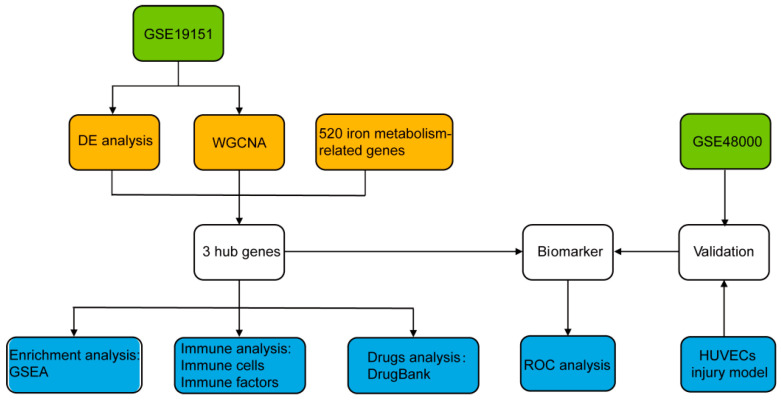
Schematic representation of the study’s workflow. The green elements signify the datasets employed for analysis and validation. Meanwhile, the orange components represent the methods used to identify hub genes. Lastly, the blue sections indicate the analytical tools applied to assess the potential and functional significance of hub genes as biomarkers.

Data on the transcriptome profile were retrieved from the Gene Expression Omnibus database, commonly abbreviated to GEO. This database encompasses two whole-blood datasets, namely GSE19151 and GSE48000. GSE19151 was made up of 133 samples, among which 63 were normal controls (NC) and 70 were VTE cases, whereas 132 samples were contained in GSE48000, with 25 NC and 107 VTE. Supplementary Table S1 summarizes the demographic information, specifically age and sex, of participants in GSE19151. A total of 520 iron metabolism-related genes, referred to as IMRGs, were collected from one previously published study [25]. Briefly, these genes were assembled by querying 14 iron metabolism-related gene sets in the Molecular Signatures Database (MSigDB), including GOBP_IRON_ION_HOMEOSTASIS, GOBP_IRON_ION_IMPORT_ACROSS_PLASMA_MEMBRANE, GOBP_HEME_METABOLIC_PROCESS, GOBP_IRON_ION_TRANSPORT, GOBP_IRON_COORDINATION_ENTITY_TRANSPORT, GOBP_CELLULAR_IRON_ION_HOMEOSTASIS, GOMF_IRON_ION_BINDING, GOMF_4_IRON_4_SULFUR_CLUSTER_BINDING, HALLMARK_HEME_METABOLISM, HEME_BIOSYNTHETIC_PROCESS, GOBP_RESPONSE_TO_IRON_ION, GOMF_2_IRON_2_SULFUR_CLUSTER_BINDING, REACTOME_IRON_UPTAKE_AND_TRANSPORT, and MODULE_540. Figure 1 showcases the comprehensive study design.

### 2.2. Differential Expression Analysis

In the research analysis of the GSE19151 dataset, we utilized the limma R package (version 3.40.6) for assessing the differential gene expression situations between the NC and VTE groups. The analysis working flow started from building gene-specific linear models through the use of the lmFit function, followed by carrying out empirical Bayes moderation by means of the eBayes algorithm. This statistical handling produced three core results: adjusted t-statistics, adjusted F-statistics, and logarithm-odds numbers for difference in expression. To define differentially expressed genes (DEGs), we built two threshold values: adjusted *p*-values which are lower than 0.05, together with absolute log2 fold change (log2FC) values which are bigger than 0.585. These standards were chosen according to settled rules for microarray data analysis, therefore guaranteeing the discovery of biologically meaningful changes in expression.

### 2.3. Weighted Gene Co-Expression Network Analysis

For the construction of a weighted gene co-expression network, we utilized the WGCNA package (version 1.73) in the R programming environment. The analysis workflow started from gene selection on the basis of median absolute deviation (MAD), in which the lower 50% of genes showing smallest expression change were removed to cut down noise disturbance. After this preprocessing step, we used the goodSamplesGenes function to further process the dataset, through deleting possible outlier genes and samples which could bring distortion to network construction. The relationships between genes were quantified through the employment of Pearson’s correlation coefficients for the construction of pairwise correlation matrices. These correlation numbers were next changed into an adjacent matrix by use of the power function Amn = |Cmn|^β, in which Cmn stands for the Pearson correlation between gene m and gene n. A soft-thresholding power (β = 8) was chosen using the analysis of network topology, because this parameter can effectively strengthen biologically meaningful strong correlations and at the same time makes the influence of weak, possibly non-functional connections smaller. The construction of the network was conducted by transforming the adjacency matrix into a topological overlap matrix (TOM) which can capture both direct and indirect gene connections. Then, average-linkage-level cluster analysis was carried out by the usage of TOM-based difference metrics for the identification of gene modules. The parameter settings of module definition were that each module must contain no less than 30 genes, and the clustering sensitivity parameter was set as 3. Module eigen genes, which stand for the leading expression mode inside every module, were computed to depict module expression profiles. Modules which show high similarity (eigengene distance < 0.25) have been merged to guarantee clear biological relevance. By means of this systematic analysis flow channel, 19 independent co-expression modules have been successfully found altogether, and kept for the following analyses.

### 2.4. Identification of Hub Genes

To accurately find core genes which are connected together with iron metabolism and VTE, we carried out cross-referencing on three gene lists. These enumerations were as follows: (i) DEGs which were obtained from the limma analysis, (ii) module genes which were obtained by WGCNA, and (iii) one pre-set group of genes which was connected with iron metabolism. We calculated the overlap of these gene lists by employing the VennDiagram package (version 1.7.3) in the R programming environment. After that, we utilized violin drawing graphs to show the differences in the expression levels of the core genes found between NC and VTE specimen groups. In order to make comparisons among different groups, statistical analysis work was done by using a two-sided *t*-test in the situation where variables showed normal distribution. In situations when normality hypotheses were not satisfied, the Mann–Whitney U test was utilized as one substitute non-parameter method. In the whole process of this research, the statistical significance was decided through the use of a threshold of *p* < 0.05.

### 2.5. Gene Set Enrichment Analysis

We performed GSEA with GSEA software (version 3.0). For each dataset, samples were stratified into high- and low-expression groups according to the average expression levels of Rho GTPase activating protein 1 (*ARHGAP1*), glucose-6-phosphate dehydrogenase (*G6PD*), and arachidonate 5-lipoxygenase (*ALOX5*), respectively. The KEGG gene set collection file (c2.cp.kegg.v7.4.symbols.gmt) was retrieved from the Molecular Signatures Database (MSigDB). Gene set size parameters were set with minimum and maximum thresholds of 5 and 5000, respectively, with 1000 permutations executed during the analysis. Gene sets were considered significantly enriched if they met the criteria of nominal *p*-value < 0.05 and false discovery rate (FDR) < 0.25.

### 2.6. Receiver Operating Characteristic (ROC) Analysis

A multivariable logistic regression model was utilized to construct the three-gene diagnostic signature. The model coefficients were estimated strictly based on the discovery dataset, yielding the following log-odds formula: Signature Score = 48.6635 − 3.7283 × (*ARHGAP1*) − 0.7206 × (*G6PD*) − 1.4600 × (*ALOX5*). To prevent overfitting and evaluate the internal robustness of the model, a 5-fold cross-validation was performed on the discovery dataset. For external validation, the coefficients established in the discovery phase were mathematically fixed. This predetermined formula was then directly applied to calculate the signature scores for patients in the independent validation cohort (dataset GSE48000), without any re-fitting or adjustment of the model weights.

It is worth noting that we processed the discovery cohort (GSE19151) and the external validation cohort (GSE48000) independently, without applying cross-dataset batch effect harmonization (such as ComBat).

The R-based pROC software package (version 1.17.0.1) was utilized for conducting these ROC analyses. Within this analytical framework, the AUC along with its corresponding confidence interval were computed using the built-in ci function of the pROC package. This methodological approach enabled quantitative assessment of how effectively the combined expression patterns of these three biomarkers could distinguish between diagnostic groups.

### 2.7. Immune Infiltration and Immune-Related Factors

CIBERSORT was performed using the LM22 leukocyte signature matrix with 100 permutations. For quality control, the CIBERSORT deconvolution *p* value was used to evaluate the reliability of immune cell fraction estimates. Samples with deconvolution *p* < 0.05 were considered reliable and were retained for the primary immune cell comparison.

Immune cell infiltration in the GSE19151 dataset was estimated via the IOBR R package (version 0.99.9). CIBERSORT was chosen as the deconvolution algorithm to assess the relative abundance scores of 22 distinct immune cell types in each sample. Furthermore, Spearman’s correlation analysis was applied to explore the associations between the expression levels of *ARHGAP1*, *G6PD*, and *ALOX5* and immune-related components. A total of 24 immunoinhibitors, 45 immunostimulators, and 41 chemokines were obtained from the TISIDB.

### 2.8. Drug Prediction from DrugBank

DrugBank (https://go.drugbank.com/, accessed on 31 May 2026) is a carefully collected drug resource which contains Food and Drug Administration (FDA)-approved agents, investigational compounds, and nutraceuticals. Therefore, to assess whether ALOX5 could act as a druggable target in a VTE situation, we carried out a query on DrugBank to find compounds that are reported to have interactions with or carry out modulation on ALOX5 activity. FDA-approved drugs targeting ALOX5 were identified based on curated drug–target annotations, followed by manual verification of the reported target–action relationships.

### 2.9. Western Blot

Human umbilical vein endothelial cells (HUVECs; iCell—h110, iCell, Shanghai, China) were cultured in Dulbecco’s Modified Eagle Medium (DMEM; SH30243.01B, Hyclone, Logan, UT, USA), which was supplemented with 10% fetal bovine serum (FBS; SH30084.03, Hyclone). Following this, the cell cultures were maintained in a humidified incubator set to 37 °C with a 5% CO_2_ environment. For the experiments, cells in the exponential growth stage were selected and randomly split into two distinct groups. In the lipopolysaccharide (LPS)-activated HUVEC model group, an endothelial inflammatory injury model was established. This was accomplished by subjecting the cells to a culture medium containing 5 µg/mL lipopolysaccharide (LPS; HY—D1056, MCE, Shanghai, China) for a duration of 24 h. In the control group, the cells were cultured in a fresh culture medium without LPS for the same 24 h period. After that, the cells from both groups were harvested. Subsequently, total protein extraction was conducted using a radioimmunoprecipitation assay (RIPA) lysis buffer (P0013B, Beyotime, Shanghai, China) that had been pre-mixed with 10% Phenylmethylsulfonyl fluoride (PMSF) (ST506, Beyotime) to inhibit protease activity. Protein concentrations were determined utilizing a Bicinchoninic acid (BCA) Protein Quantification Kit (P0010, Beyotime) following the manufacturer’s standard protocol. After concentration normalization, the protein samples were subjected to heat denaturation at 95 °C to ensure complete unfolding of protein structures. Equal amounts of denatured proteins were then separated by sodium dodecyl sulfate-polyacrylamide gel electrophoresis (SDS-PAGE) and electrotransferred onto polyvinylidene fluoride (PVDF) membranes (IPVH00010, Millipore, Boston, MA, USA). The membranes were first blocked with a 5% non-fat milk solution prepared in Tris-buffered saline with Tween 20 (TBST) for 60 min at room temperature to minimize non-specific antibody binding. Following blocking, the membranes were incubated overnight at 4 °C with specific primary antibodies, including anti-ALOX5 (10021-1-Ig, Proteintech, Wuhan, China; 1:1000 dilution) and anti-GAPDH (60004-1-Ig, Proteintech; 1:5000 dilution) as an internal reference. After three rounds of washing with TBST to remove unbound primary antibodies, the membranes were incubated with corresponding secondary antibodies for 1 h at ambient temperature. The secondary antibodies used were anti-ALOX5 (111-035-003, Jackson, Lancaster, PA, USA) and anti-GAPDH (115-035-003, Jackson), both diluted at 1:10,000. Protein bands were visualized using enhanced chemiluminescence (ECL) detection reagent (NCI5079, Thermo, Waltham, MA, USA) and imaged using a gel documentation system. The relative intensities of the protein bands were quantified using ImageJ software (version 1.53k, National Institutes of Health, Bethesda, MD, USA), with GAPDH serving as the loading control for the normalization of protein expression levels. LPS-activated HUVECs were used as an in vitro endothelial inflammatory injury model to evaluate ALOX5 protein expression under inflammatory endothelial activation. This model reflects one cellular component of VTE-related thrombo-inflammation, but does not reproduce the full pathophysiological complexity of VTE.

## 3. Results

### 3.1. Hub Gene Identification Integrating VTE-Associated Signals and Iron Metabolism

**Figure 2:**
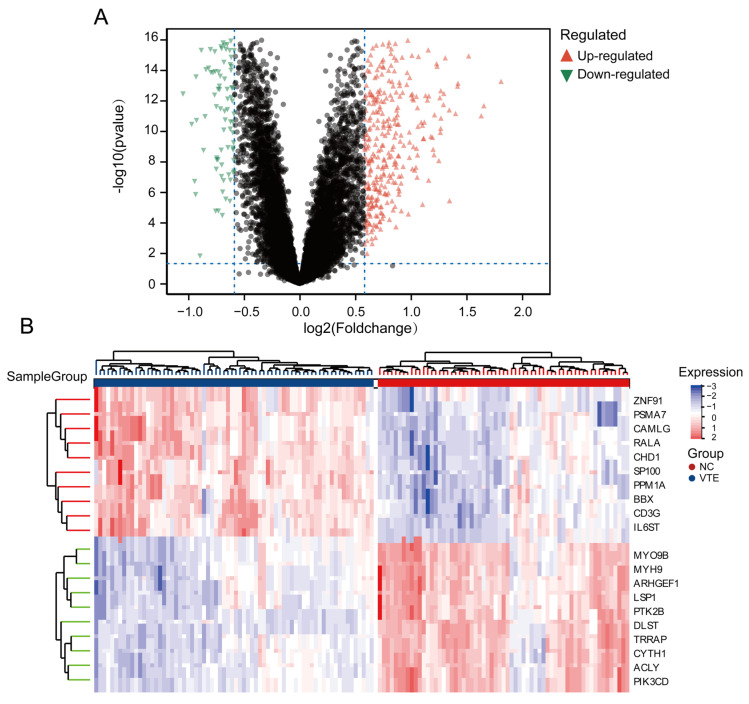
Depiction of the DEGs which lie between VTE and NC samples. In part (**A**), the total results are given visually. Red dot points mean genes which have obviously higher expression quantity in VTE samples, and green dot points are those that have obviously increased expression in NC samples. In order to emphasize the most important changes, a heatmap (**B**) has been made by us. This heatmap is used to show the expression situations of the first 20 genes which have the biggest difference in expression when we compare the disease group (VTE) with the control group (NC).

To start our research work, we characterized transcriptomic differences between VTE and NC specimens in the GSE19151 dataset. Through the use of already-built filtering standards (|log2 fold change| > 0.585 and adjusted *p*-value < 0.05), this analysis has obtained an overall total of 457 genes that have differences in expression. Among these, 335 transcription products displayed lifted expression situations while 122 presented lowered expression in VTE specimens when put beside comparison groups (Supplementary Table S2). The total distribution of DEGs is outlined in a volcano plot (Figure 2A), and the 10 genes with the most increase and the 10 genes with the most decrease are shown by visualization in a heatmap (Figure 2B).

**Figure 3:**
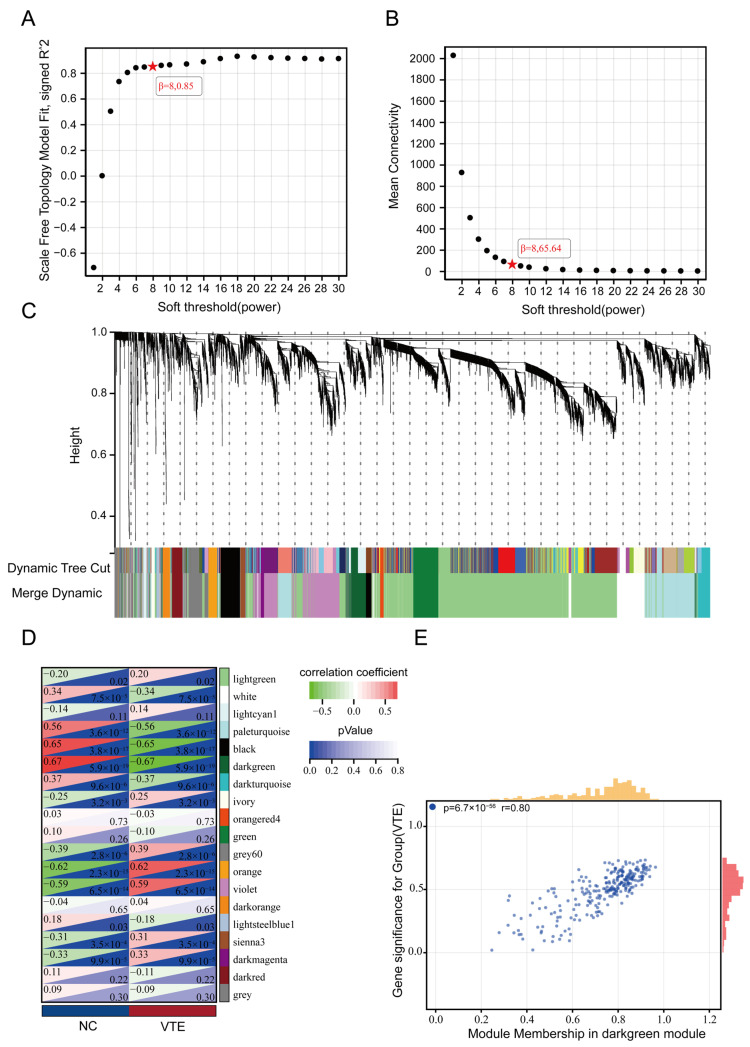
WGCNA was performed on the GSE19151 dataset. The upper left panel (**A**) illustrates the relationship between the scale-free fit index (y-axis) and the soft-thresholding power (β, x-axis), providing critical parameters for network construction. Adjacent to this, Panel (**B**) demonstrates how average connectivity (y-axis) varies with different soft-thresholding power values (β, x-axis), aiding in the selection of optimal network parameters. Moving to the lower portion, Panel (**C**) presents a hierarchical clustering dendrogram of the detected genes, where distinct co-expression modules are visually distinguished by color coding in the row beneath the dendrogram structure. Panel (**D**) displays a correlation heatmap between module eigengenes (rows) and clinical characteristics (columns), with each cell containing both the correlation coefficient and its corresponding statistical significance (*p*-value). Finally, Panel (**E**) provides a scatterplot analysis that correlates gene significance for a key clinical trait with module membership values across all genes within the dark green module, facilitating the identification of biologically relevant gene modules.

In order to catch coordinated expression programs which exceed single-gene changes, we carried out WGCNA on GSE19151 after quality control had been completed. Overall, 12,823 genes that cover 133 samples were retained for constructing the network, and therefore we chose a soft-thresholding power of value 8. Therefore, this network achieved approximate no-scale characteristic behavior with a scale independence degree equal to 0.85 and an average connection degree of 65.64 (Figure 3A,B). For the identification of co-expression modules, we utilized the dynamic tree cutting algorithm, which uses parameter settings with a minimum module size of 30 and deepSplit value of 3. After we finished first-step module searching, we therefore put further modules together on the basis of eigengene unlikeness, hence setting the combining threshold value to 0.25. This method of analysis brought about the finding of 19 different co-expression modules (Figure 3C). The correlation analysis between traits and modules indicated that the dark green module is the one that has the strongest connection with VTE status (r = −0.67, *p* = 5.9 × 10^−19^; Figure 3D), hence indicating that its whole expression mode was conversely connected with VTE. Therefore, the dark green module is given priority to be used for downstream screening work. As the result shows, the connection between GS and MM inside this module indicates that the intramodular connectivity and phenotype relevance have very close correspondence (r = 0.80, *p* = 6.7 × 10^−56^; Figure 3E), and the entire gene list is given in Supplementary Table S3.

**Figure 4:**
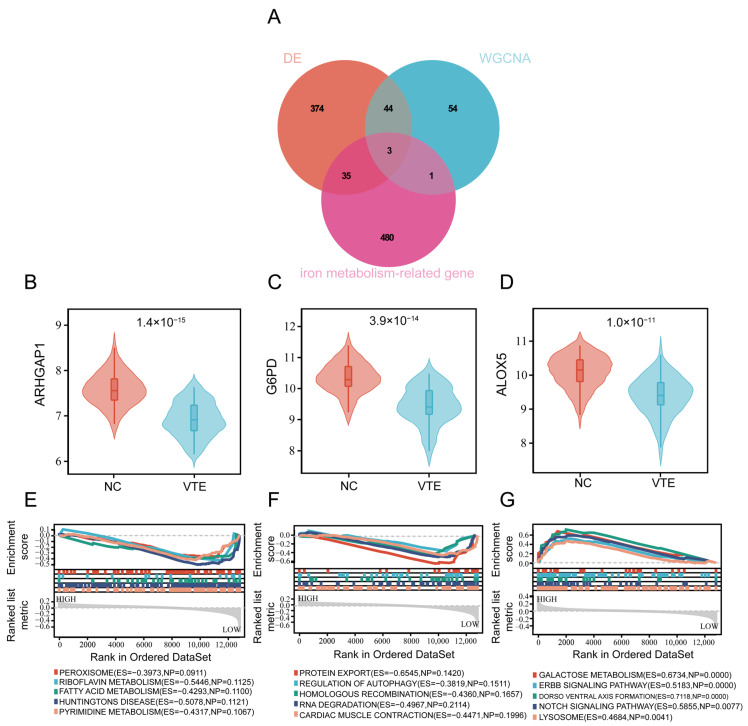
Findings of GSEA and hub gene analysis. Identification of hub genes (**A**): Venn diagram was used to illustrate the overlap among three gene sets: DEGs between VTE and NC samples, genes from the dark green module of WGCNA, and IMRGs. Through this analysis, *ARHGAP1*, *G6PD*, and *ALOX5* were pinpointed as the hub genes. Comparison of Hub Gene Expression: Violin plots were employed to examine the expression levels of these hub genes in VTE and NC samples from the GSE19151 dataset. Specifically, the plots for *ARHGAP1* (**B**), *G6PD* (**C**), and *ALOX5* (**D**) were generated to visually represent the differences in their expression. GSEA Results for Hub Genes: GSEA was conducted to identify the pathways enriched by the expression of these hub genes. The results for *ARHGAP1* (**E**), *G6PD* (**F**), and *ALOX5* (**G**) show the pathways associated with their expression.

Finally, we integrated three complementary gene sets—DEGs (*n* = 457), dark green module genes (*n* = 103), and iron metabolism-related genes (*n* = 520)—and identified three overlapping candidates: *ARHGAP1*, *G6PD*, and *ALOX5* (Figure 4A). Violin plots showed that all three genes were significantly downregulated in VTE compared with NC in GSE19151 (Figure 4B–D). Their expression patterns in the validation dataset GSE48000 are presented in Supplementary Figure S1.

### 3.2. Biological Pathways Associated with the Hub Genes

Next, we explored the biological background of these hub genes by means of GSEA. Therefore, *ARHGAP1* and *G6PD* could not achieve statistical significance for pathway enrichment under the applied standards, whereas consistent associations between *ALOX5* and multiple VTE-related pathways are displayed by it.

Specifically, *ALOX5* was linked to endothelial function and vascular remodeling pathways, including VEGF SIGNALING PATHWAY (Normalized Enrichment Score (NES) = 1.5341, False Discovery Rate (FDR) = 0.0856), RENIN ANGIOTENSIN SYSTEM (NES = 1.6280, FDR = 0.0569), and VASOPRESSIN REGULATED WATER REABSORPTION (NES = 1.4995, FDR = 0.1053). Enrichment was also observed in pathways related to cytoskeletal dynamics and cell–matrix/cell–cell adhesion, such as REGULATION OF ACTIN CYTOSKELETON (NES = 1.7007, FDR = 0.0347), FOCAL ADHESION (NES = 1.5286, FDR = 0.0874), and ADHERENS JUNCTION (NES = 1.6794, FDR = 0.0398). In addition, metabolic programs emerged, including GALACTOSE METABOLISM (NES = 2.0751, FDR = 0.0033), GLYCEROLIPID METABOLISM (NES = 1.7084, FDR = 0.0334), and ADIPOCYTOKINE SIGNALING PATHWAY (NES = 1.7525, FDR = 0.0304). Notably, several immune- and inflammation-related pathways were enriched as well, including LEUKOCYTE TRANSENDOTHELIAL MIGRATION (NES = 1.8648, FDR = 0.0303), FC GAMMA R MEDIATED PHAGOCYTOSIS (NES = 1.8065, FDR = 0.0242), LYSOSOME (NES = 1.8384, FDR = 0.0220), and CHEMOKINE SIGNALING PATHWAY (NES = 1.5686, FDR = 0.0719) (Figure 4G). Taken together, these results indicate that reduced ALOX5 expression tracks with pathways governing endothelial regulation, immune cell recruitment/activation, platelet-relevant adhesion architecture, and lipid metabolism—mechanistic axes that are highly congruent with current concepts of VTE pathobiology. Full enrichment statistics are provided in Supplementary Table S4.

### 3.3. Performance of a Hub Gene-Based Diagnostic Signature

**Figure 5:**
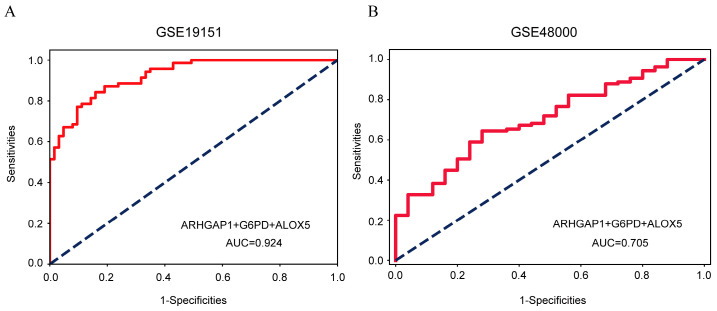
ROC curves have been utilized to evaluate the diagnostic capability of *ARHGAP1*, *G6PD*, and *ALOX5* when distinguishing VTE from NC samples, and the corresponding AUC values are given for two independent datasets: GSE19151 (**A**) and GSE48000 (**B**).

We have done an evaluation on whether the expression quantities of *ARHGAP1*, *G6PD*, and *ALOX5* can assist in distinguishing VTE from NC samples. The ROC analysis that was conducted on the discovery dataset (GSE19151) obtained an AUC value of 0.924, which shows that the diagnostic performance is strong. When we carried out tests on the independent cohort GSE48000, this model obtained an AUC of 0.705 (Figure 5). This cross-data-set verification proves that it is possible to use *ARHGAP1*, *G6PD*, and *ALOX5* as a transcriptomic mark which has possible clinical worth for VTE screening.

### 3.4. Immune Infiltration Landscape and Immune-Related Correlates

The thrombotic space is formed through mutual actions between the vessel endothelium, platelet pieces, and immunity cells—especially neutral granular cells and single-core cells—inside a fibrin/extra-cell-material support frame and under the effect of inflammation medium substances and blood solidification factors. This interface between immunity and coagulation (immunothrombosis) may promote the generation of NETs, strengthen the stability of thrombi, and regulate the process of fibrinolysis and the reconstruction of venous walls, hence influencing the initiation, resolution, and recurrence risk of thrombi. Accordingly, the remodeling of immunity is therefore a plausible path for finding biomarkers and doing therapeutic targeting in VTE.

**Figure 6:**
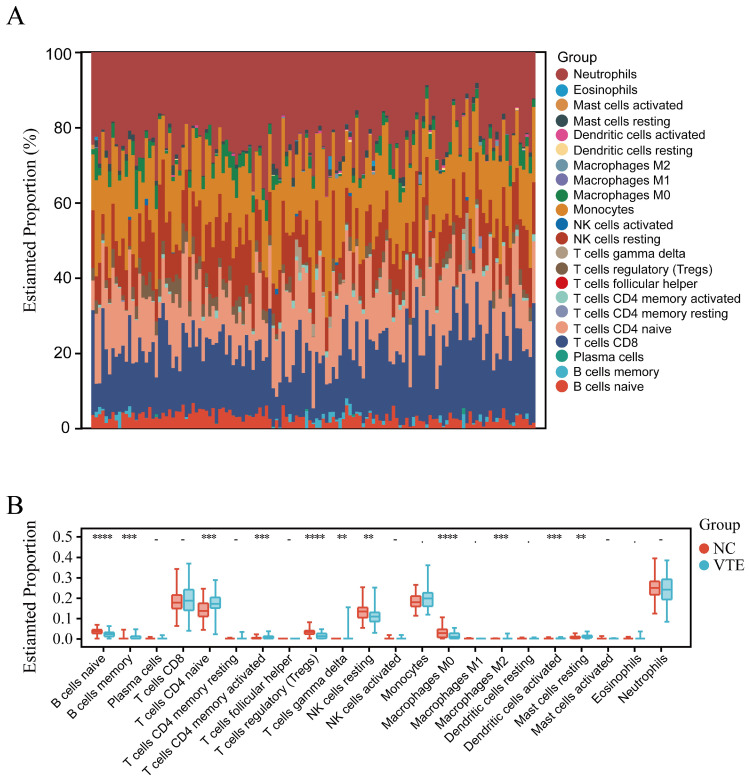
Analysis of immune cell infiltration. (**A**) A heatmap is presented which depicts the relative prevalence of 22 distinct immune cell types within each individual sample. (**B**) A violin plot is used to contrast the infiltration scores of each immune cell type between the VTE and NC groups. ** *p* < 0.01, *** *p* < 0.001, **** *p* < 0.0001. The symbol “-” indicates no statistical significance (*p* ≥ 0.05), and “.” indicates marginal significance (*p* < 0.1).

By means of CIBERSORT, we have completed the estimation of relative component proportions of 22 immune cell groups among 70 VTE samples and 63 NC samples (Figure 6A). Comparative analysis has discovered that the proportions of memory B cells (*p* = 7.4 × 10^−4^), naive CD4 T cells (*p* = 2.0 × 10^−4^), activated memory CD4 T cells (*p* = 6.6 × 10^−4^), gamma delta T cells (*p* = 6.0 × 10^−3^), M2 macrophages (*p* = 1.5 × 10^−4^), activated dendritic cells (*p* = 9.8 × 10^−4^), and resting mast cells (*p* = 1.5 × 10^−3^) are obviously higher in VTE. In contrast, naive B cells (*p* = 2.9 × 10^−6^), regulatory T cells (Tregs) (*p* = 8.7 × 10^−9^), resting NK cells (*p* = 3.4 × 10^−3^), and M0 macrophages (*p* = 8.7 × 10^−5^) were significantly decreased in VTE (Figure 6B).

**Figure 7:**
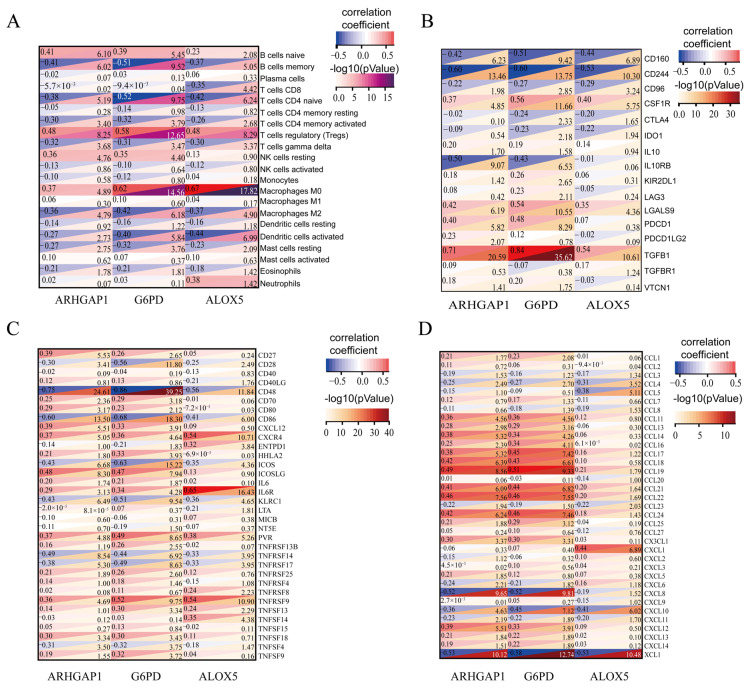
The present study conducted a systematic correlation analysis examining the association between the expression patterns of *ARHGAP1*, *G6PD*, and *ALOX5* genes and various immune-related biomarkers. Visualized through comparative graphs, the analysis explores the connection between mRNA expression levels of these three genes and multiple immunological parameters, including the following: (**A**) infiltration density scores and phenotypic characteristics of distinct immune cell populations, (**B**) transcriptional profiles of key immunoinhibitory molecules, (**C**) expression patterns of critical immunostimulatory factors, and (**D**) secretion levels of major chemokine families. Each comparative analysis incorporates statistical validation, with both Spearman correlation coefficients and corresponding *p*-values reported to establish the significance of observed relationships.

Given that *ALOX5* showed the most prominent functional signal in pathway analysis, we further examined its immune associations. Correlation heatmaps summarize relationships between *ALOX5* expression and immune cell infiltration as well as immunoinhibitors, immunostimulators, and chemokines (Figure 7A–D). Among the 22 immune cell types, *ALOX5* expression was significantly correlated with 13 subsets (Figure 7A). Positive correlations were observed with naive B cells, Tregs, M0 macrophages, and neutrophils, whereas negative correlations were observed with memory B cells, CD8 T cells, naive CD4 T cells, activated memory CD4 T cells, gamma delta T cells, M2 macrophages, activated dendritic cells, resting mast cells, and eosinophils; the strongest association was with M0 macrophages. These patterns suggest that *ALOX5* may be linked to coordinated changes in both innate and adaptive immune infiltration in VTE.

It should be noted that the interpretation of CIBERSORT-inferred macrophage subsets in peripheral blood requires caution. True tissue macrophages are not expected to be abundant in circulating blood samples. Therefore, the “M0 macrophage” fraction estimated by CIBERSORT in this context should not be interpreted as direct evidence of increased circulating macrophages. Rather, it likely represents a monocyte/macrophage-lineage-related transcriptional program or an undifferentiated myeloid-like signal captured by the LM22 reference matrix.

With respect to immune-regulatory molecules, 8 of 16 immunoinhibitors were significantly associated with *ALOX5* expression, with *TGFB1* showing the most significant relationship, followed by CD244 and CD160 (Figure 7B). Among 36 immunostimulators, 18 had obvious statistical correlation with *ALOX5*; *IL6R* showed the most powerful association, with *CD48*, *TNFRSF9*, and *CXCR4* following behind (Figure 7C). Among 35 chemokines, 14 presented marked correlation with *ALOX5*; *XCL1* was the top related factor, and *CXCL1* and *CXCL10* came next (Figure 7D). Therefore, these research results support the theory that *ALOX5* may have a potential function in constructing the immune microenvironment of VTE, because it has joint connection relations with immune cells and immune-related factors.

### 3.5. DrugBank-Derived Candidate Compounds Targeting ALOX5

To research treatment feasibility, we conducted an inquiry on DrugBank and found 19 approved drugs marked to target ALOX5 (Table 1). These medicines were mainly inhibitors, with a small number being categorized as potentiators or substrates.

### 3.6. Dysregulated Expression of ALOX5 in the HUVECs Injury Model

**Figure 8:**
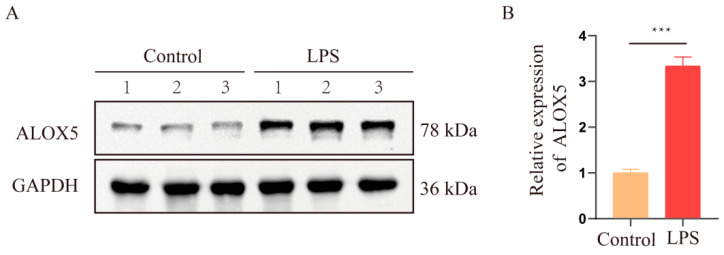
Relative expression analysis of ALOX5 within the HUVECs injury cell model. (**A**) Western blot examination was carried out to assess the expression of ALOX5 in both the control group and the group treated with LPS. Glyceraldehyde-3-phosphate dehydrogenase (GAPDH) was employed as an internal loading control to standardize protein expression levels across samples. To ensure statistical reliability, three independent biological replicates were loaded for each experimental group. (**B**) Quantitative analysis of protein band intensity was conducted using ImageJ software to determine the relative expression levels of ALOX5 in the injury model group compared to control samples. Experimental data are expressed as the mean ± standard deviation (SD), where *** indicates a statistically significant difference with a *p*-value < 0.001.

To further clarify the possible function of ALOX5 in VTE’s occurrence and development, we built an inflammatory damage model by treating HUVECs with LPS. We measured the protein expression level of ALOX5 through Western blot analysis. The results showed that ALOX5 expression had obvious upregulation in the LPS-treated group when comparing with untreated control HUVECs (*p* < 0.001; Figure 8). Therefore, these discoveries confirm that ALOX5 expression is not normal during inflammatory injury of vascular endothelial cells; hence, this situation hints at ALOX5’s possible use as a biomarker for VTE, and thus offers cellular-level experimental proof to support its being considered as a potential therapeutic target.

## 4. Discussion

The clinical relevance of iron metabolism in VTE has gained increasing attention in recent years. Accumulating epidemiological and clinical evidence has established a strong association between iron deficiency, anemia, and an elevated risk of VTE. Specifically, a recent systematic review highlighted anemia as a potent yet frequently underrecognized driver of VTE [26]. Furthermore, large-scale retrospective studies and clinical observations have consistently demonstrated that both iron deficiency and iron deficiency anemia are significantly associated with an increased incidence of pulmonary embolism and deep vein thrombosis [27,28]. These macroscopic clinical observations perfectly align with our molecular findings, reinforcing the hypothesis that dysregulated iron homeostasis is not merely a bystander, but actively participates in the thrombo-inflammatory pathogenesis of VTE.

Iron may also contribute to VTE pathobiology through immunometabolic regulation. Beyond its classical function in erythropoiesis, iron participates in mitochondrial metabolism, redox balance, cell proliferation, cytokine production, and immune cell effector responses. Cronin et al. reported that altered iron homeostasis can reshape immune cell metabolism and contribute to immune-related diseases [29]. Because VTE involves thrombo-inflammatory interactions among endothelial cells, leukocytes, platelets, and the coagulation system, iron-mediated immunometabolic alterations may provide a plausible biological context for the iron metabolism-related gene signature identified in this study.

In this research work, we establish an integrative transcriptomic workflow for the purpose of discovering genes linked to iron metabolism—genes that have associations with VTE—and further assess their diagnostic capability among separate patient cohorts. Therefore, by combining differential expression analysis and WGCNA, we move away from the single-gene screening method to a more all-around approach, concentrating on co-expression modules which have close connections to the VTE (Vascular Endothelial System) status. Hence, the overlapping part of DEGs, the leading module related to VTE, and a chosen group of iron metabolism-related genes identifies three hub genes, *ARHGAP1*, *G6PD*, and *ALOX5*, all of which demonstrated downregulation within the discovery dataset. A signature composed of three genes selected from the candidate pool demonstrated strong discriminatory ability in the training dataset and retained its accuracy in an external validation group, indicating its applicability across different cohorts.

The identified hub genes encode proteins with distinct but potentially complementary functions in VTE pathobiology. *ALOX5* encodes 5-lipoxygenase, an iron-dependent enzyme involved in leukotriene biosynthesis and inflammatory lipid mediator production. *ARHGAP1* encodes a regulator of Rho family GTPase signaling, which participates in cytoskeletal remodeling, endothelial barrier regulation, cell adhesion, and leukocyte migration. *G6PD* encodes the rate-limiting enzyme of the pentose phosphate pathway and contributes to NADPH generation, antioxidant defense, and redox homeostasis. Therefore, these hub genes may reflect the coordinated involvement of lipid inflammation, endothelial/immune-cell migration, and metabolic-redox imbalance in VTE.

Among the three hub genes, *ALOX5* has the most consistent functional associations. In our GSEA, *ARHGAP1* and *G6PD* did not show statistically significant pathway enrichment. However, *ALOX5* demonstrated a strong association with endothelial and vascular signaling pathways, including the VEGF and renin–angiotensin systems. It was also linked to cytoskeletal organization processes, such as actin filament regulation and adhesion junction formation. Additionally, *ALOX5* was involved in metabolic pathways, particularly carbohydrate and lipid metabolism, as well as immune-inflammatory responses, including leukocyte migration and chemokine signaling. These processes correspond to recognized mechanisms in VTE, encompassing endothelial activation or dysfunction, recruitment of immune cells, and the coupling of metabolic and inflammatory pathways [30,31,32,33].

ALOX5 may also represent a link between iron metabolism, lipid-mediated inflammation, and immune crosstalk in VTE. *ALOX5* encodes arachidonate 5-lipoxygenase, an iron-dependent enzyme involved in leukotriene biosynthesis and inflammatory cell activation. Notably, Lu et al. recently identified ALOX5 as a potential therapeutic target for tubulointerstitial inflammation in diabetic kidney disease using integrated machine learning, multi-omics analysis, and WGCNA. Because diabetic kidney disease is associated with chronic metabolic inflammation, endothelial dysfunction, and increased thromboembolic risk, this external evidence supports the broader relevance of ALOX5 in immune-metabolic and thrombo-inflammatory disease contexts. However, VTE-specific functional validation remains necessary [34].

Immune remodeling is another key feature of the VTE transcriptome. Immune-cell deconvolution using the CIBERSORT method showed significant changes in various leukocyte subsets, suggesting that peripheral blood transcriptomes are indicative of systemic immune disturbances in VTE [33,35]. Additionally, *ALOX5* expression is positively correlated with the numbers of naïve B cells, regulatory T cells, M0 macrophages (monocyte/macrophage-lineage-related signal), and neutrophils, but negatively correlated with memory B cells and specific T-cell subsets. The most striking connection we have observed is related to M0 macrophages (monocyte/macrophage-lineage-related signal). This result is in line with existing academic materials, which lay strong stress on the function of monocyte/macrophage programs in both thrombus growth and thrombus resolution [14,36]. Therefore, these research findings provide support to the “immunothrombosis” framework. The framework puts forward the theory that myeloid and lymphoid lineages together influence thrombus biology via cytokine secretion, the interaction between endothelial cells and immune cells, and the regulation of fibrinolysis [37].

At the cellular and molecular levels, the functional crosstalk between iron metabolism and ALOX5 (5-lipoxygenase) is particularly notable in macrophages, which are key drivers of thrombo-inflammation in VTE. ALOX5 is a lipid-metabolizing enzyme whose catalytic activity strictly relies on a non-heme iron atom. Importantly, recent functional evidence has demonstrated that intracellular iron availability directly orchestrates the subcellular trafficking and activation of ALOX5 in human macrophages [38]. Alterations in iron homeostasis can therefore dynamically regulate ALOX5-dependent lipid mediator production and macrophage activation. Furthermore, the targeted interference of ALOX5 has been shown to significantly alleviate inflammatory responses and profibrotic changes under metabolic stress conditions [39]. In the context of VTE, these functional links provide compelling orthogonal evidence suggesting that iron dysregulation may promote a pro-inflammatory macrophage phenotype via the modulation of ALOX5, thereby exacerbating endothelial injury and immunothrombosis.

It is noteworthy that while transcriptomic detection of full-blood specimens in VTE displayed lowered *ALOX5* expression, our LPS-activated HUVEC model presented raised the protein amount. This disparity is possibly brought about by differences in sample composition and inflammatory background. Whole blood is a complex mixture of various cell types, with myeloid cells as the primary ALOX5 producers. Systemic thrombosis may cause these myeloid cells to be consumed or move out of blood flow, thus leading to a general decrease in ALOX5 transcript levels [40,41]. In contrast, endothelial cells respond to LPS stimulation via activation of the NF-κB pathway, which then enhances the production of inflammatory mediators and eicosanoids. ALOX5 has an extremely important function in the synthesis of these eicosanoids [40,42]. Previous studies have verified that endothelial cells can generate bioactive lipids through ALOX family members under both normal and abnormal body conditions. These results together indicate that the observed opposite trends reflect region-specific and time-associated regulation of ALOX5, emphasizing its dual roles in systemic and endothelial inflammatory–thrombotic processes.

It should be emphasized that the LPS-activated HUVEC system used in this study is not a complete experimental model of VTE. As summarized by Ayyoub et al., thrombosis models include a broad range of in vivo and in vitro systems, and in vivo venous thrombosis models are generally required to reproduce the integrated effects of venous stasis, blood flow alteration, coagulation activation, platelets, leukocytes, endothelial cells, and vascular wall responses [43]. In contrast, in vitro endothelial cell models are more suitable for dissecting specific cellular and molecular events involved in thrombo-inflammatory activation. Therefore, we used LPS-activated HUVECs not to mimic the entire VTE process, but to evaluate whether ALOX5 is responsive to inflammatory endothelial injury, a key component of VTE pathobiology.

From the translation application perspective, our DrugBank search found several approved drugs targeting ALOX5, with zileuton being a prominent example. Although database labeling alone cannot confirm the clinical significance in VTE, studies in vascular biology demonstrate that pharmacological modulation of 5-lipoxygenase activity may affect endothelial inflammatory activation and vascular reactivity [44,45,46]. Subsequent research should evaluate whether ALOX5-targeting interventions can alter the interactions between endothelial cells, immune responses, and coagulation processes, or influence thrombus resolution in preclinical VTE models.

Several limitations should be noted. First, our diagnostic markers were constructed from public microarray datasets, which may involve cross-platform differences and sampling inconsistencies. Second, immune cell profiling relied on computational deconvolution. Due to the current unavailability of public VTE single-cell RNA-seq (scRNA-seq) cohorts, orthogonal validations via flow cytometry or de novo scRNA-seq are required to definitively map the cell-type-specific regulatory landscape. Third, the discordant expression of ALOX5—downregulated in bulk peripheral blood but upregulated in LPS-stimulated HUVECs—highlights its complex, cell-type-specific regulation. While our HUVEC model supports ALOX5’s role in localized endothelial inflammation, it does not resolve its regulation in circulating myeloid cells. Therefore, further investigations incorporating primary human leukocytes, platelets, and in vivo thrombosis models are necessary to establish exact cause–effect relationships. Last but not least, future prospective studies must confirm the clinical value of this marker and assess its incremental predictive capability compared to existing VTE predictors.

To conclude, we have identified and conducted external verification on a three-gene signature related to iron metabolism, that is, *ARHGAP1*, *G6PD*, and *ALOX5*; this signature is used to distinguish VTE, and we connected it to endothelial, metabolic, and immune processes. Among them, *ALOX5* showed robust functional and immunologic associations and exhibited marked expression changes in an inflammatory endothelial injury model. These findings prove that *ALOX5* acts as a potential biomarker and a key mechanistic link in the context of VTE, and provides a basis for further exploration into the relationship between iron metabolism and immune interactions in the context of thrombus biology.

## Figures and Tables

**Table 1:** Drugs targeting ALOX5 obtained from the DrugBank database.

Gene Symbol	Protein	UniProt ID	Name	Drug Group	Actions
*ALOX5*	5-lipoxygenase	P09917	alpha-Tocopherol succinate	approved, nutraceutical, vet_approved	other/unknown
			Aminosalicylic acid	approved, investigational	inhibitor
			Balsalazide	approved	inhibitor
			Cannabidiol	approved, investigational	inhibitor
			D-alpha-Tocopherol acetate	approved, nutraceutical, vet_approved	other/unknown
			Diacerein	approved, vestigational, withdrawn	inhibitor
			Diclofenac	approved, investigational, vet_approved	potentiator
			Diethylcarbamazine	approved, investigational, vet_approved, withdrawn	inhibitor
			Fostamatinib	approved, investigational	inhibitor
			Huperzine A	approved, investigational, withdrawn	inhibitor
			Ibuproxam	approved, withdrawn	inhibitor
			Icosapent	approved, investigational, nutraceutical	substrate
			Meclofenamic acid	approved, investigational, vet_approved	inhibitor
			Mesalazine	approved, investigational	inhibitor
			Minocycline	approved, investigational	inhibitor
			Montelukast	approved, investigational	other/unknown
			Omega-3 fatty acids	approved, investigational, nutraceutical	substrate
			Sulfasalazine	approved, investigational	inhibitor
			Vitamin E	approved, investigational, nutraceutical, vet_approved	other/unknown
			Zileuton	approved, withdrawn	inhibitor

## Data Availability

The data presented in this study are openly available in the article.

## Supplementary Materials

The following supporting information can be downloaded at https://www.mdpi.com/article/10.3390/cimb48060607/s1.
