# Supplementary material for: Transcriptomic Profiling and WGCNA Identify ALOX5 as a Key Regulator of Iron Metabolism and Immune Crosstalk in Venous Thromboembolism
Source: Curr Issues Mol Biol. 2026 Jun 10;48(6):607. doi: 10.3390/cimb48060607 (PMC13297702; doi:10.3390/cimb48060607)

Figure S1. Validation of the expression levels of three hub genes in the GSE48000 dataset.
(A) The expression level of ARHGAP1 in the NC and VTE groups.
(B) The expression level of G6PD in the NC and VTE groups.
(C) The expression level of ALOX5 in the NC and VTE groups.

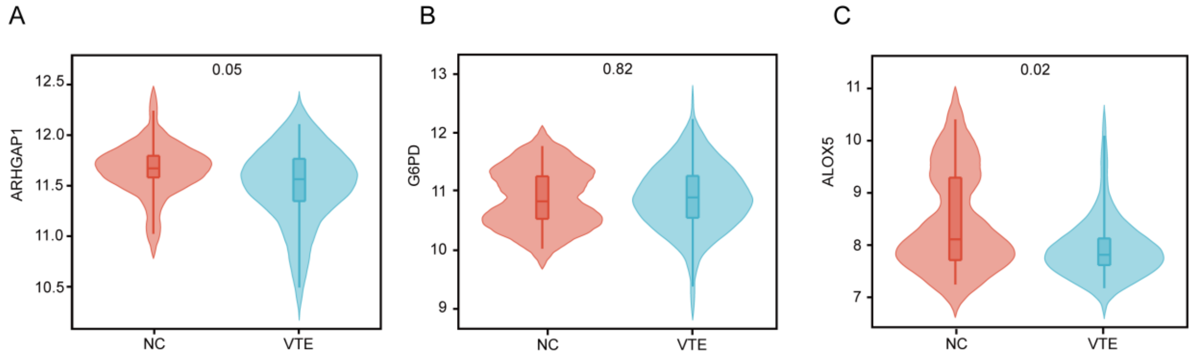

Supplement: Supplementary file 1 [file cimb-48-00607-s001.zip › Supplementary Figures S1-Expression of ARHGAP1 (A), G6PD (B) and ALOX5 (C) in GSE48000 dataset.pdf]
