# Supplementary material for: Transcriptomic Profiling and WGCNA Identify ALOX5 as a Key Regulator of Iron Metabolism and Immune Crosstalk in Venous Thromboembolism
Source: Curr Issues Mol Biol. 2026 Jun 10;48(6):607. doi: 10.3390/cimb48060607 (PMC13297702; doi:10.3390/cimb48060607)
Supplement: Supplementary file 1 [file cimb-48-00607-s001.zip › Supplementary Table S1-Demographic characteristics of VTE cases and healthy controls (NC) in the GSE19151 dataset.pdf]

Supplementary Table S1. Demographic characteristics of VTE cases and healthy controls (NC) in the GSE19151 dataset.

GSE19151

	NC Group (n=63)	VTE Group (n=70)	*p*
**Age,y**			
mean	27.5	50.6	1.32E-17
Range	18-56	23-84	
**Gender,n**			
Men	34 (54.0%)	27 (38.6%)	
Women	29 (46.0%)	43 (61.4%)	
